# Oral squamous cell carcinoma – do we always need elective neck dissection? evaluation of clinicopathological factors of greatest prognostic significance: a cross-sectional observational study

**DOI:** 10.3389/fonc.2023.1203439

**Published:** 2023-09-14

**Authors:** Adam Michcik, Adam Polcyn, Maciej Sikora, Tomasz Wach, Łukasz Garbacewicz, Barbara Drogoszewska

**Affiliations:** ^1^ Department of Maxillofacial Surgery, Medical University of Gdansk, Gdańsk, Poland; ^2^ Department of Maxillofacial Surgery, Hospital of the Ministry Interior, Kielce, Poland; ^3^ Department of Maxillofacial Surgery, Medical University of Łódź, Łódź, Poland

**Keywords:** oral squamus cell carcinoma, floor of the mouth, tongue cancer, prognostic factors, elective neck dissection, depth of invasion, grading

## Abstract

The aim of the study was to find clinical and pathological factors with the greatest prognostic significance in patients with OSCC. The analysis included 125 patients grouped according to the tumor primary site (TPS): the floor of the mouth (FOM), tongue (TC) and retromolar triangle (RMT). Grading (G), tumor size (pT), nodal metastases (NM), local recurrence (LR), nodal recurrence (NR), perineural invasion (PNI), lymphovascular invasion (LVI), extranodal extension (pENE), and nodal yield (NY) were evaluated in each group.

**Results:**

With regard to TPS, FOM appeared to be the most metastatic. However, the recurrence rate was similar to TC tumors, which were characterized by higher G than those in other locations. When analyzing G, the highest percentage of LR (40.5%) and NM (34.5%) was observed among patients with G2. As G increased, so did the number of pENE G1 – 7.4%; G2 – 31%; G3 – 35.7%; LVI: G1 – 25.9%; G2 – 50%; G3 – 57.1%; PNI: G1 – 29.6%; G2 – 47.6%; G3 – 92.9%; NR G1 – 14.8%; G2 – 32.1%; G3 – 21.4%. Grading did not affect the type of growth and did not directly affect the occurrence of NR. pT and DOI increased the frequency of NM but we did not observe any effect of pT and DOI on LR, PNI, and LVI. NY in the study group did not increase the risk of NR.

**Conclusion:**

Tumor primary sites within the FOM, TC, and pT classification are the factors that increase the risk of NM and LR. However, apart from the primary site predisposing to the occurrence of NM, the histological structure of the tumor turned out to be the most important feature affecting the patient’s prognosis. The number of cases of pENE+, LVI+, PNI+, NM+, and NR+ increased with the increase in G. Although the pT, DOI increased the frequency of NM, we did not observe the effect of the pT and DOI on LR, PNI and LVI. Thus, even in the case of a small tumor of the FOM and TC with at least G2, elective neck dissection should be performed each time.

## Introduction

Oral squamous cell carcinoma is the 6th most common cancer (1) and accounts for over 90% of oral cancers (2, 3).

Despite advances in medicine, surgical techniques and our knowledge of tumor biology, surgeons are still unable to improve the 5-year survival rates of patients with OSCC (oral squamous cell carcinoma). Survival rates of 48% (1, 3) are not satisfactory (4).

The increasing number of patients in the third and fourth decades of life necessitates a deeper understanding of the biology of OSCC (5, 6). Low public awareness, lack of oncological vigilance among primary care physicians and dentists and disregard for the problem by the patients themselves result in high rates of patients presenting to specialized care at an advanced stage. Moreover, the incidence of OSCC has increased over the past two decades, to 0.7% and 1.8% for men and women, respectively (7). The risk of local, nodal recurrence (LR/NR), and the 5-year survival rate depend on many factors (3, 8).

Information in the available literature points to pT – the tumor size, G – grading, or TPS – the tumor primary sites as the prognostically significant features (9–11).

The occurrence of perineural invasion (PNI) may be related to the chemotropism of neoplastic cells (12) and is not specific to OSCC only (13, 14). Its occurrence, similarly to lymphovascular invasion (LVI), is an unfavorable prognostic factor (15–17). In addition to the above, another unfavorable prognostic factor is extranodal extension (pENE), and its occurrence is associated with a possible increase in the risk of nodal recurrence (NR) (18, 19); however, a comparison of these features, along with an assessment of the TPS, DOI and the occurrence of nodal or local metastases (NM/LR), nodal recurrence NR and G will give us a better understanding of what combination of the aforementioned factors may be associated with the most unpredictable course of the disease. It should be noted that patients often die as a result of the locoregional spread of the tumor to the lymphatic system (20) rather than as a result of the growth of the primary tumor. Metastasis is a common feature of malignant tumors (21, 22). In addition, due to the primary site of the tumor in the oral cavity, in which numerous areas with a rich lymphocytic lining are located, the risk of NM is high (9, 23).

Nevertheless, some patients with small pT1 OSCC have positive N+ cervical lymph nodes or develop recurrence within a short time after their treatment, while others, sometimes even with larger primary tumors, have neither metastatic lymph nodes nor recurrences. Due to the unsatisfactory results of treatment, it has been attempted to find and identify the prognostic factors, both improving and deteriorating the prognosis.

An extensive multivariate retrospective analysis may contribute to a better understanding of the biology of the tumor and, thus, the clinical course of the disease. This is all the more important upon a clear tendency toward a younger age of diagnosis of patients with OSCC (5, 6, 11, 24, 25). Studies indicate a higher survival rate in younger age groups (26), but it still remains at an unsatisfactory level.

For this reason, the authors set themselves the task of comparing OSCC features such as TPS in the oral cavity, G, pT, DOI (measured in mm), PNI, LVI and pENE. Additional comparison of the cases of LR and NR (confirmed within 2 years of surgical treatment), or NM (confirmed by post-surgical histopathological examination) allowed for an assessment of the factors worsening the patient’s prognosis. Based on detailed clinical data collected before and after the surgery, statistical analysis was performed to determine which tumor features have a significant impact on the patient’s prognosis.

## Materials and methods

The analysis included 125 patients diagnosed with OSCC, treated in the Department of Maxillofacial Surgery of the Medical University of Gdańsk in the years 2017-2019. The patients were in the 5th-8th decade of life, median 64.4 years. The average age was 64.4 years, 67.2% were males.

All of the patients were smokers for at least 5 pack-years and consumed alcohol 10-50g/day.

Non-smokers were excluded due to a very small number of cases.

Additional factors taken into account in the selection of patients for the study group are the occurrence of distant metastases M and high-risk HPV infection.

Patients with M were excluded from the study – they were not qualified for surgical treatment. In most cases, they were referred for systemic oncological treatment (radiochemotherapy). Single cases of generalized patients treated surgically and by metastasectomy were not eligible for the study group.

The objective of the study was to determine the factors that increase the occurrence of NM without specifying the positive lymph node level. NM is the most significant aggravating factor in the prognosis of patients with OSCC. Therefore, the study determined the overall presence or absence of NM.

The qualified patients with a p16-negative result were divided according to the TPS into the floor of the mouth (FOM), tongue cancers TC and the retromolar triangle RMT groups. Postoperative histopathological results were evaluated for the following features: pT, DOI two groups: DOI > 10 mm and DOI ≤ 10 mm), G, PNI, LVI, pENE, nodal yield (NY), nodal metastasis (NM) and occurrence of LR and NR within 2 years after the surgery.

The eligible patients underwent radical tumor resection (R0 margins) and had the least elective neck dissection END (I-III) in the case of radiographic N0 n = 74, nodal yield NY – END $\bar{x}$ = 23.9 (patients with FOM and TC tumor were subjected to a bilateral nodal surgery), while in the case of radiographic N+ or the intraoperative finding of metastatic lymph nodes, modified radical neck dissection RMND (I-V) was performed (n = 51), nodal yield NY – RMND $\bar{x}$ = 32.1 either unilaterally or bilaterally for carcinoma of the FOM and TC. Patients with postoperatively confirmed NM, including pENE+ or/and DOI > 10 mm and PNI+ received postoperative radiotherapy (PORT). Adjuvant treatment according to the Dahanc scheme was instituted in n = 68 cases. The areas were treated according to risk with 50 to 60 Gy in 33 fractions 5 or 6 times a week.

None of the patients received neoadjuvant radiotherapy. The follow up time for local and nodal recurrences was 2 years (**Table 1**).

**Table 1:** Characteristics of the study group; 8^th^ TNM classification used; legends: TC, tongue cancer; FOM, floor of the mouth; RMT, retromolar triangle; TPS, tumor primary site; G, grading; pT, tumor size; DOI, depth of invasion; NM, nodal metastases; LR, local recurrence; NR, nodal recurrence; pENE, extranodal extension; PNI, perineural invasion; LVI, lymphovascular invasion; ND, neck dissection; END, elective neck dissection; RMND, radical modified neck dissection; KPS, Karnofsky performance score.

Characteristic		N	%
Gender	male female	84 41	67.2 32.8
Age	50^th^ 60^th^ 70^th^ 80^th^	6 73 42 4	4.8 58.4 33.6 3.2
Smoking	No< 5 pack- years ≥ 5 pack-years	not qualified not qualified 125	- - 100
Alcohol intake	< 10 g/day 10-30 g/day 31- 50g/day > 50 g/day	- 52 73 1	- 41.6 58.4 0.8
TPS	TC FOM RMT	30 55 40	24.0 44.0 32.0
G	G1 G2 G3	27 84 14	21.6 67.2 11.2
pT	pT1 pT2 pT3 pT4	31 49 28 17	24.8 39.2 22.4 13.6
DOI	≤ 10mm > 10 mm	69 56	55.2 44.8
NM	No Yes	89 36	71.2 28.8
LR	No Yes	84 41	67.2 32.8
NR	No Yes	91 34	72.8 27.2
pENE	No Yes	92 33	73.6 26.4
PNI	No Yes	64 61	51.2 48.8
LVI	No Yes	68 57	54.4 45.6
ND	END RMND	74 51	59.2 40.8
KPS	80-90 50-70	80 45	64 36

Using the obtained data, the incidence of NM and/or LR and NR was calculated according to TPS, G, pT, DOI, pENE, PNI, LVI, and NY. The influence of clinicopathological factors on the recurrence and survival was evaluated. Fisher’s test and McNemar’s test were used in the analysis. For quantitative evaluation, t-student test was implemented in the case of normal distribution, and Mann-Whitney (two-sided Wilcoxon sum test) in the cases presenting non-normal distribution. Analysis was also conducted by using variance tests (for more than 2 classes): one-way ANOVA – in the case of a normal distribution and the Kruskal-Wallis rank-sum test – in the case of a distribution that does not belong to the normal distribution.

## Results

The study showed that not all the evaluated factors proved to be prognostic, with statistically significant differences between them. However, we were able to isolate those whose preoperative and postoperative assessments may influence our decisions to continue a patient’s treatment. In the first stage of the study, the TPS was evaluated in terms of the distribution of G, the occurrence of NM, or LR. In the case of TC, it was evident that tumors with higher grading (G3 – 35.7%, G2 – 23.8%, G1 – 18.5%, p.value = 0.499) were more common than in other TPS, while G2 tumors were most common in the FOM (72.1%, p.value = 0.033). **Table 2**. Next, we analyzed whether TPS influenced the incidence of NM and/or LR. The results indicated that cancers located in the FOM metastasized most frequently (32.45%, p.value = 0.428 Fisher test) in the evaluated group (n = 55) of patients, which was also observed by other authors.

**Table 2:** Distribution (n/%) of G and LR according to the OSCC primary site.

TPS		FOM (n = 68)	TC (n = 30)	RMT (n = 40)
G(n)	G1 G2 G3	16 (23.5%) 49 (72.1%) 3 (4.4%)	5 (16.7%) 20 (66.7%) 5 (16.7%)	5 (16.7%) 25 (62.5%) 6 (15.0%)
LR	No (n = 84) Yes (n = 41)	43 (63.2%) 25 (36.8%)	19 (63.3%) 11 (36.7%)	31 (77.5%) 9 (22.5%)

TPS, tumor primary site; LR, local recurrence; FOM, floor of the mouth; TC, tongue cancer, RMT, retromolar triangle.

Among patients with TC tumors (n = 30), NM were found in 26.7% (p.value = 0.822), while in those with RMT (n = 40, p.value = 0.03) it was 20%. The development of LR within 2 years after surgical treatment was confirmed in 41 cases. Furthermore, divided according to the TPS, the percentage distribution was as follows: among patients with TC and FOM, LR was found in 36.7% (p.value = 0.658) and 36.8% (p.value = 0.343), respectively. In contrast, among patients with OSCC of the RMT, LR occurred in 22.5% of cases (p.value = 0.106). **Table 2**.

In the second stage, we evaluated whether G affects the incidence of LR and NR, NM, pENE, DOI, LVI and PNI. Among patients with OSCC G1, LR was reported in 22.2%, n = 6 cases, G2 – 40.5% n = 34, G3 – 7.1% n = 1, p.value = 0.018. The result is statistically significant and yields OSCC G2 as the subtype with the highest risk of LR. On the other hand, histopathological assessment confirmed NM in the G1 group of patients, metastasis was confirmed in 11.1% n = 3 patients, G2 – 34.5% n = 29, and G3 – 28.6% n = 4; p.value = 0.058. The results indicate that similarly to LR, NM was also most frequently found in the G2 variant of the tumor. When evaluating the effect of the G score on the risk of pENE, it was found that the incidence of pENE increased with the increasing G of the tumor, p.value = 0.024; pENE+: G1 – 7.4%; n = 2; G2 – 31%, n = 26; G3 – 35.7%, n = 5.

G did not affect the type of growth (endophytic or exophytic) in the groups evaluated. The distribution of G was similar; DOI ≤ 10 mm: G1 – 21.74%, G2 – 66.67%, G3 – 11.59%; DOI > 10 mm: G1 – 21.43%, G2 – 67.86%, G3 – 10.71%, which may suggest that the type of tumor growth does not depend on histological G.

The distribution of LVI and PNI in the individual G groups was as follows: LVI: G1 – 25.9%, n = 7; G2 – 50%, n = 42; G3 – 57.1%, n = 8; p.value = 0.058; PNI: G1 – 29.6%, n = 8; G2 – 47.6%, n = 40; G3 – 92.9%, n = 13; p.value = 0.00034. The distribution of PNI among group G turned out to be statistically significant. However, no statistical significance was noted when assessing the incidence of NR among the individual G groups.

NR in the G groups was G1 – 14.8%, n = 4; G2 – 32.1%, n = 27; G3 – 21.4%, n = 3; p.value = 0.214. **Table 3**.

**Table 3:** Distribution (n/%) of LR, NM, pENE+, DOI ≤ 10 mm, DOI > 10mm, LVI, PNI, and NR according to G.

		G1	G2	G3
LR		6(22.2%)	34(40.5%)	1(7.1%)
NM		3(11.1%)	29(34.5%)	4(28.6%)
pENE+		2(7.4%)	26(31%)	5(35.6%)
DOI	≤10mm>10mm	12 (21.74%) 15 (21.43%)	38 (66.67%) 46 (67.86%)	6 (11.59%) 8 (10.71%)
LVI		7(25.9%)	42(50%)	8(57.1%)
PNI		8(29.6%)	40(47.6%)	13(92.9%)
NR		4(14.8%)	27(32.1%)	3(21.4%)

Due to the common perception that DOI is a prognostically significant factor, an attempt was made to evaluate whether the DOI affects the incidence of LR, NM, PNI and LVI.

Statistical analysis showed no increase in the incidence of LR with increasing DOI; moreover, a slightly higher LR rate of 37.7% was found in the group of patients with invasion ≤ 10mm, compared to 26.8% in the group with invasion > 10mm, (p.value = 0.251). This result is quite surprising, and the available literature provides scarce reports investigating the DOI with regard to the risk of LR.

**Figure 1:**
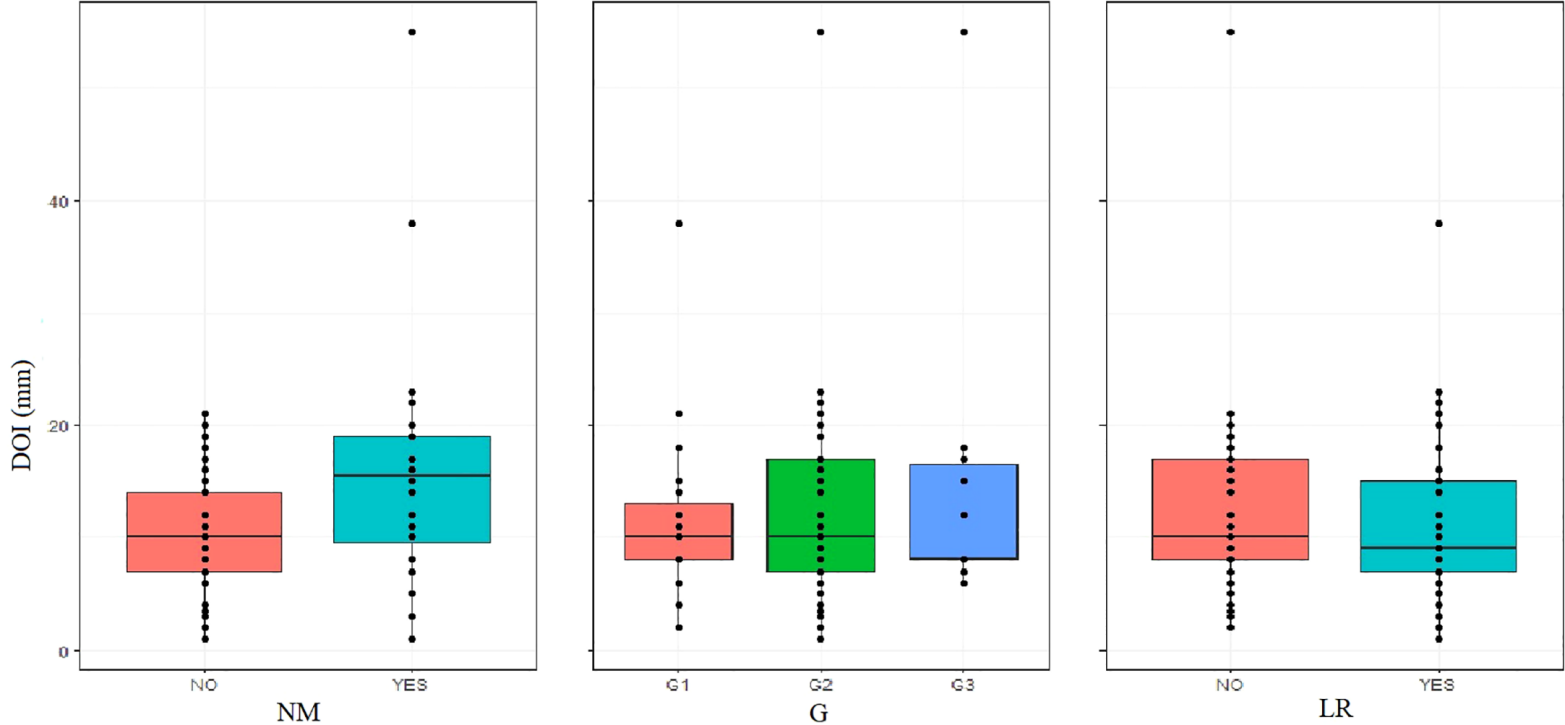
Distribution of DOI as quantitative data regarding NM, G and LR.

Nonetheless, when correlating DOI with confirmed NM, a higher incidence of NM in patients with DOI > 10mm – 41.1%, n = 13 was observed in comparison with the group ≤ 10mm – 18.5%, n = 23. This difference was statistically significant (p.value = 0.009, Wilcoxon rank sum test with continuity correction, p.value< 0.05), which allows us to conclude that patients with tumors with endophytic growth type and DOI > 10mm have a higher risk of NM, which, as we know, significantly worsens the prognosis of the patients and reduces 5-year survival rate (27, 28) as well as qualifies the patient for postoperative complementary radiotherapy (29, 30), **Figure 1**.

The analysis of the frequency of PNI and LVI in the individual DOI groups was not statistically significant, and the distribution was as follows; PNI: DOI ≤ 10mm – 42%, n = 29; DOI > 10mm – 57.1%, n = 32; p.value = 0.1074; LVI: DOI ≤ 10mm – 42%, n = 29, DOI > 10mm – 50%, n = 28; p.value = 0.4703.

**Figure 2:**
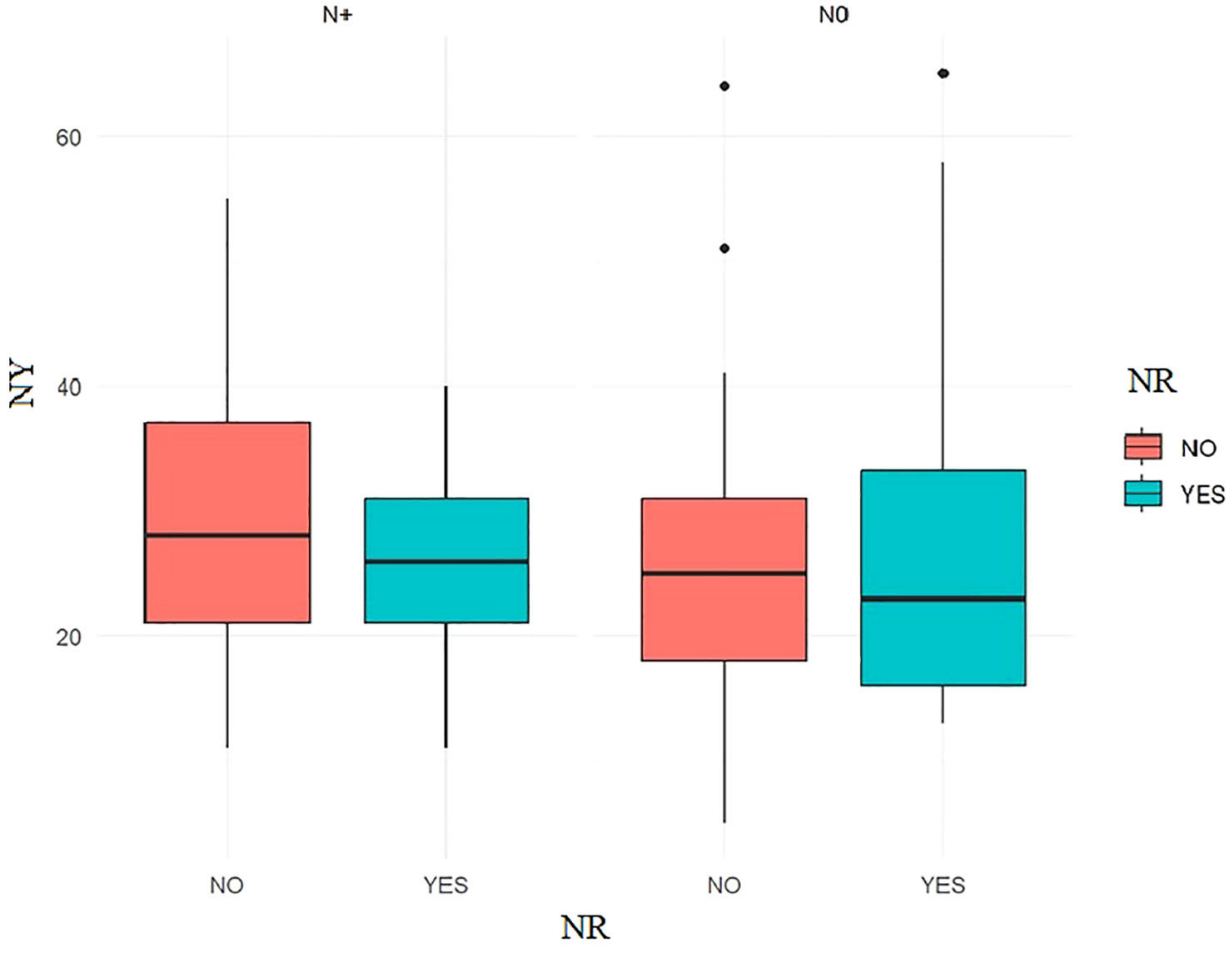
The Welch Two Sample t-test, NY grouped by the presence of NR showed that no significant differences between the groups could be observed: p.value ≥ 0.05.

Nodal yield is an important prognostic factor. The average number of lymph nodes removed in the entire study group was 27 (elective neck dissection END nodal field $\bar{x}$ = 23.9; modified radical Neck dissection MRND $\bar{x}$ = 32.1). Dividing patients into two groups in terms of the surgical treatment of the neck lymphatic system: radiological N0 – END and radiological N+ – MRND, no statistically significant differences between the study groups could be demonstrated. **Figure 2**.

Then, it was assessed whether NR was more common among patients diagnosed with pENE.

Among patients without pENE – NR occurred in 20.7%, n = 19, while in the pENE+ group, NR occurred in 45.5%, n = 15; p.value = 0.01 (statistically significant differences can be observed).

The last parameter evaluated was pT. No statistically significant results were found upon evaluation of the incidence of LR in the studied groups: pT1 – 25.8%, n = 8; pT2 – 40.8%, n = 20; pT3 – 28.6% n = 8; pT4 – 29.4%, n = 5; p.value = 0.514. However, when assessing the incidence of NM, a correlation was observed.

**Figure 3:**
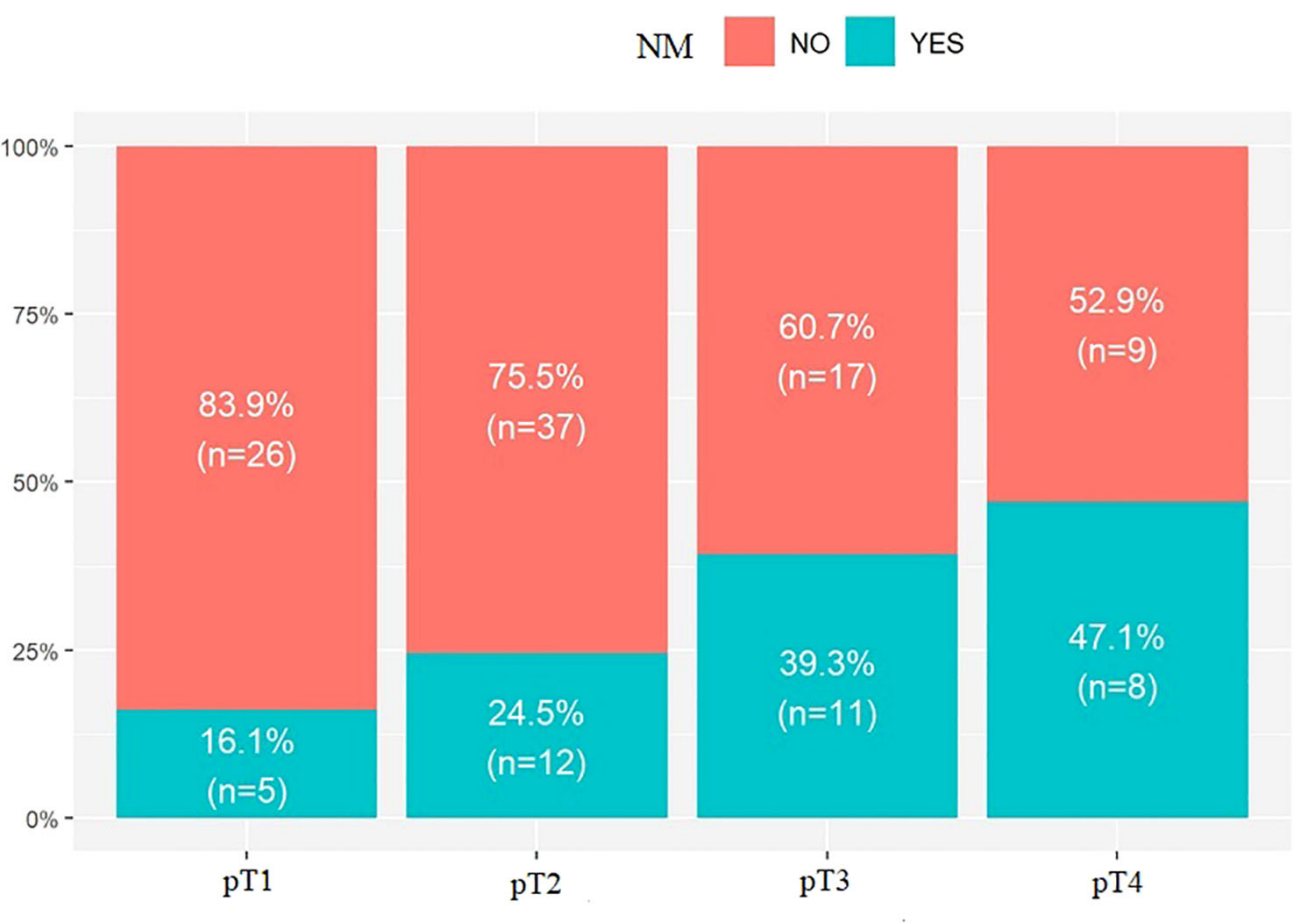
Percentage distribution of NM relative to pT. p.value = 0.067.

An increase in NM with pT was observed: pT1 – 16.1%, n = 5; pT2 – 24.5%, n = 12; pT3 – 39.3%, n = 11; pT4 – 47.1%, n = 8; p.value = 0.067; **Figure 3**, **Table 4**.

**Table 4:** Summary of characteristic values (* statistical significance – p.value ≤ 0.05).

		G				NM			LR		
		G1 (n)	G2 (n)	G3 (n)	p.value	Yes (n=36)	No (n=89)	p.value	Yes (n=41)	No (n=84)	p.value
TPS	FOM (n=68)	16	49	3	0.584	22	46	0.428	25	43	0.343
	TC (n=30)	5	20	5	0.499	8	22	0.822	11	19	0.658
	RMT (n=40)	9	25	6	0.033*	8	32	0.203	9	31	0.106
G	G1 (n=27)	-	-	-	-	3	24	0.058	6	20	0.018
	G2 (n=84)	-	-	-	-	29	55		34	51	
	G3 (n=14)	-	-	-	-	4	10		1	13	
pT	pT1 (n=31)	9	18	4	0.204	5	26	0.067	8	23	0.514
	pT2 (n=49)	13	33	3		12	37		20	29	
	pT3 (n=28)	3	19	6		11	17		8	20	
	pT4 (n=17)	2	14	1		8	9		5	12	
DOI	≤10mm (n=69)	15	46	8	1	13	56	0.009*	15	41	0.251
	>10mm (n=56)	12	38	6		23	33		26	43	

## Discussion

The increasing number of cases of squamous cell carcinoma is a global health problem (31). The identification of prognostic factors affecting patients’ 5-year survival is of utmost importance and may contribute to a better understanding of the biology and clinical course of cancer, as well as influence surgical treatment standards.

It seems important to identify clinical as well as histopathological parameters of the tumor, which will determine the extent of treatment and/or its type. The analysis carried out highlighted the tumor characteristics that affect the increased risk of NM or LR and NR, which significantly affect the 5-year survival of patients (27, 32).

Therefore, the decision determining the scope of treatment, including neck dissection (ND), must be based on a broad analysis of the evaluated prognostic factors. The aim of the authors was to determine which of them is the most important and should have the greatest impact on our therapeutic decisions. In addition, broad-spectrum analysis provided an answer which combination of the factors has the greatest impact on deterioration of the prognosis.

The G of the patients who underwent surgical treatment was assessed according to the WHO guidelines. However, it should be noted that there are also other assessment methods, e.g., Trojani and Coindre’s three-stage division of histological malignancy was based on three tumor characteristics: 1. similarity to mature tissue, 2. extent of necrosis, 3. number of mitoses per 10 fields of view. It is a prognostically significant indicator (33, 34). The result of the study reveals that TC tumors have more commonly a higher G than cancers in other primary sites. Additionally, the G2 subtype was found to be the most metastatic. The results show that TC and FOM tumors have the highest risk of LR, 36.7% (p.value = 0.658) and 36.8% (p.value = 0.343), respectively, while NM metastases were most common in the group of patients with FOM cancer (32.45%, p.value = 0.428 Fisher test).

This result confirms the information available in the literature that the rich lymphatic lining of the floor of the mouth favors the formation of NM (35).

No publications that would jointly compare the TPS in terms of LR and NM were found in the available literature. Our study demonstrates such a correlation. However, it should be noted that only the 3 most common OSCC locations were assessed. Others require further analysis. In addition to TPS, other factors that have a prognostic impact were taken into account and assessed.

G is considered as an important prognostic factor; a study by Doshi et al. shows a direct correlation between the occurrence of NM depending on G (36). Khawaja et al. believe that tumor G should be taken into account when selecting the appropriate treatment method (37).

Shu Ting Chuang et al., on the other hand, suggest that even for small ≥ G2 tumors, END or adjuvant radiotherapy is advised (38). Moreover, he considers the G2 variant of the tumor as an important factor in worsening the patient’s prognosis (39), which was also confirmed in our study.

Among the analyzed patients, NM and LR were most frequently found in the group of patients with G2. As is well known, regional spread is a poor prognostic factor (35), and its occurrence significantly reduces the patient’s prognosis. FOM tumors were proved to be the most metastatic in the studied group.

The authors of the present study, like Chairat Burusapat et al., point out the necessity of performing END in the majority of patients with OSCC of the TC, FOM (40).

In a retrospective study conducted between 1991 and 2017, Wichmann G. et al. demonstrated the correlation between the increase in the survival rate and the increase in the number of performed ENDs (41).

LR is associated with several factors, such as pT, TPS, G, PNI, and LVI. The tumor resection itself is also extremely important, and it should be noted that all operated patients qualified for analysis in the postoperative histopathological result had R0 margins. The resulting n = 41 puts oral cancers among those with a high risk of LR. It should be noted that the G obtained during the patient’s pre-procedural diagnosis (biopsy) can provide us with a lot of valuable information.

As shown by the experience, the OSCC G level affects the risk of PNI and LVI, which are important prognostic factors (15, 16, 20, 42).

Our study, therefore, showed that the higher the G, the higher the patient’s risk of PNI (p.value = 0.00034) and LVI (p.value = 0.058) as well as pENE (p.value = 0.024). There was no direct relationship between the increase in NR and G.

The occurrence of NR was influenced by the patient’s primary nodal status (N+) and the presence of pENE. We showed that the presence of pENE significantly increases the risk of NR (p.value = 0.01).

Similar conclusions from studies available in the literature place pENE as a significant risk factor for NR (18, 43, 44).

A direct relationship between pENE and NR demonstrated in this study indicates the need to qualify patients with pENE+ to the high-risk group of NR.

The endophytic type is the most common type of OSCC growth. In the study group, as many as 44.8% of patients had a tumor with a DOI > 10mm. However, an increased frequency of higher G and larger number of recurrences among these patients (DOI > 10mm) were not observed. In contrast, the results of other authors indicate an increased risk of LR with increasing DOI. Farhan Zubair et al. state that DOI > 10mm is predictive of an increased risk of LR (45). This fact shows how unpredictable OSCC is as a neoplasm, which makes it very difficult to develop gold standards for therapeutic approaches.

The DOI can be found in the literature as a very important prognostic factor in OSCC (4, 32, 46, 47). DOI is often associated with an increased risk of NM (48, 49). Moreover, the 8th American Joint Committee on Cancer (AJCC) included the DOI in the criteria for assessing the size of the tumor T, which confirms the extremely important prognostic role of the DOI (50).

A similar result was obtained in our study. Available publications indicate that DOI can be a clinical tool for predicting hidden NM and determining the need for END in the early stages of OSCC (51) and PORT (52).

In our study, we have shown that DOI is a very important factor in determining the risk of locoregional dissemination of cancer, which often determines treatment failures in patients with OSCC.

The distribution of G in the particular DOI groups was almost identical, which indicates that the G does not affect the type of tumor growth.

However, we did not find a direct relationship between the increase in the incidence of PNI and LVI with the increase in DOI, and the differences between the selected DOI groups in relation to the LVI and PNI characteristics were not statistically significant. On the other hand, it should be remembered that the tumor G is one of the most important factors affecting the occurrence of LVI and PNI.

In his study, Harri Keski-Säntti confirmed that the DOI of a tumor affects the incidence of NM. In a retrospective study, he analyzed the occurrence of NM and diagnosed it in 24% of patients with pT1 cancer and 35% of those with pT2 (53).

The above result is confirmed by the analysis conducted and described in our study as well as others available in the literature (54, 55).

The aforementioned data indicates the necessity of performing END in every patient with at least G2 grade FOM and TC tumors.

In a comprehensive study encompassing 372 cases of OSCC recurrences, L. J. Oh explicitly notes that patients who underwent END accounted for 15.1% (n = 98) of the studied group, while the remaining 274 recurrences were patients who had undergone surgery without END (41.5%) (54).

Nodal yields are a prognostic factor increasing the risk of NR, and the number of at least 18 lymph nodes removed during a surgical procedure is associated with improved patient survival and lower rates of NR (56, 57).

In the study group of patients, the average NY was for END NY $\bar{x}$ = 23.9; MRND NY $\bar{x}$ = 32.1. Such a result translated into a lack of statistical significance in the studied groups, and thus no effect of NY on NR was found.

Discussing the obtained results, we can conclude that the histological structure of the tumor is the most important feature affecting the occurrence of factors worsening the prognosis, and its determination must be an essential element of planning the scope of the surgery (including END) and adjuvant treatment. Clinical decisions in the treatment process should be based in the first place on the histological structure of the tumor and then on its pT and location.

Due to the increasing incidence of OSCC, especially in younger age groups (1, 5, 6), a deeper understanding of OSCC biology is extremely important. The presented study has clearly identified the factors deteriorating the prognosis most significantly, which is extremely important in view of the global cancer problem.

## Conclusions

The study identified the factors that worsen the prognosis of patients with OSCC TPS of the FOM and TC with the type of endophytic growth (DOI > 10mm) and at least G2 turned out to be the most disseminated locoregionally and prognostically unfavorable. A high incidence of NM and LR, known to be a very unfavorable prognostic factor, was observed in this group (55, 58).

However, apart from the location predisposing to the occurrence of NM, the histological structure of the tumor (G) was found to be the most important feature affecting the patient’s prognosis.

The number of cases of pENE+, LVI+, PNI+, NM+ and NR+ increased with G, although the pT, including the DOI parameter, increased the frequency of NM, but we did not observe the effect of pT, including DOI, on LR, PNI and LVI.

Based on the obtained results, we can put forward the thesis that the risk of high DOI is not dependent on the level of tumor grading G. Apart from the factors deteriorating the patient’s prognosis, it has been proven that the G value of a tumor does not affect its type of growth.

It should also be emphasized that patients with pENE+ features must be automatically included in the group with the highest risk of NR.

The NY of 18, described many times in the literature, is, according to many authors, a number below which there is an increased risk of NR. The result above 18 obtained in our study did not increase the risk of NR, which confirms the above thesis.

The obtained result revealed a group of OSCC prognostic factors whose presence should always oblige the surgeon to perform END, also in the case of radiological N0. Even small tumors of the FOM and TC with at least G2 and endophytic type of growth (DOI) must be resected with simultaneous END.

## Author contributions

Conceptualization AM. Methodology AM, AP. Software TW, LG. Validation AM, MS. Formal analysis AM, AP. Investigation AM. Resources AM, AP, MS, TW, BD. Data curation AM, AP. Writing—original draft preparation AM. Writing—review and editing MS, BD. Visualization AM, LG, TW. Supervision AM, MS. Project administration AM, MS, BD. All authors contributed to the article and approved the submitted version.
